# Collet-Sicard Syndrome Secondary to Tuberculosis of the Base of the Skull: A Case Report

**DOI:** 10.7759/cureus.73920

**Published:** 2024-11-18

**Authors:** Siddhant Jain, Ravi Talapa, Nidhi Yadav, Anjali Chanyal, Kunal Saini

**Affiliations:** 1 Internal Medicine, Atal Bihari Vajpayee Institute of Medical Sciences and Dr. Ram Manohar Lohia Hospital, New Delhi, IND

**Keywords:** collet-sicard syndrome, diabetes mellitus complication, jugular foramen syndrome, lower cranial nerve palsy, skull base tuberculosis, steroid therapy

## Abstract

Collet-Sicard syndrome, resulting from the involvement of all four lower cranial nerves, is an extremely rare condition. This case report details a 69-year-old female patient who presented with classic signs and symptoms of lower cranial nerve palsies (IX, X, XI, and XII) and was subsequently diagnosed with Collet-Sicard syndrome secondary to tuberculosis at the base of the skull. A contrast-enhanced MRI of the neck revealed bone marrow edema in the clivus, occipital condyle, and C1 vertebra, along with diffuse surrounding soft tissue swelling and collection, findings consistent with tuberculosis. The patient was treated with antitubercular therapy and steroids, along with neuromuscular and vocal rehabilitation. She showed significant improvement two months after starting antitubercular therapy and steroids. Tubercular Collet-Sicard syndrome should be suspected in patients presenting with cranial nerve palsies, elevated erythrocyte sedimentation rate, and abnormal imaging, as early recognition and treatment can lead to successful recovery.

## Introduction

The involvement of all four cranial nerves (IX through XII) results in Collet-Sicard syndrome with all findings ipsilateral to the site of injury. The syndrome is characterized by paralysis of the trapezius and sternocleidomastoid muscles, paralysis of the vocal cord and pharynx, hemiparesis of the tongue, loss of taste on the posterior one-third of the tongue, and hemianesthesia of the palate, pharynx, and larynx [1]. The main causes include neoplasms of the skull base, carotid artery dissections, and basilar skull fractures. Tuberculosis leading to Collet-Sicard syndrome has never been documented before. The condition should be suspected in regions where tuberculosis is highly prevalent as timely diagnosis and treatment with antitubercular therapy can result in successful recovery. This case highlights an unusual presentation of tuberculosis manifesting as Collet-Sicard syndrome.

## Case presentation

History

A 69-year-old female with a 25-year history of diabetes mellitus (managed on regular insulin) and hypertension presented with a history of gradually progressive difficulty in swallowing, initially with solid food for the past three months and later with both solids and liquids for the past one month. She also complained of hoarseness of voice, slurred speech, headache over the occipital area, and dizziness for the past two months. She reported a significant weight loss (10 kg) over the past six months. She had no history of fever, cough, shortness of breath, decreased hearing, or deviation of the angle of mouth. She had no prior history of tuberculosis or head and neck trauma. She had undergone a left-sided mastoidectomy for otitis media, which was uneventful.

Clinical examination

On examination, her blood pressure was 128/70 mmHg, pulse rate was 78/minute, and oxygen saturation (SpO2) was 98% on room air. Her random blood glucose was 324 mg/dL, with a glycosylated hemoglobin (HbA1c) of 13.8%. There were no significant abnormalities on general physical examination. Cranial nerve examination revealed right-sided lower motor neuron palsy of cranial nerves IX, X, XI, and XII. Findings included nasal regurgitation of food, a nasal twang to her voice, drooping of the palate to the right, deviation of the uvula to the left, and a depressed right-sided gag reflex. Weakness of the sternocleidomastoid was noted on the right side, but no weakness of the trapezius was observed. There was hemiatrophy of the tongue with deviation toward the right. Meningeal signs were negative, and the rest of the neurological examination was normal. Other systemic examinations revealed no abnormalities.

Investigations

**Figure 1:**
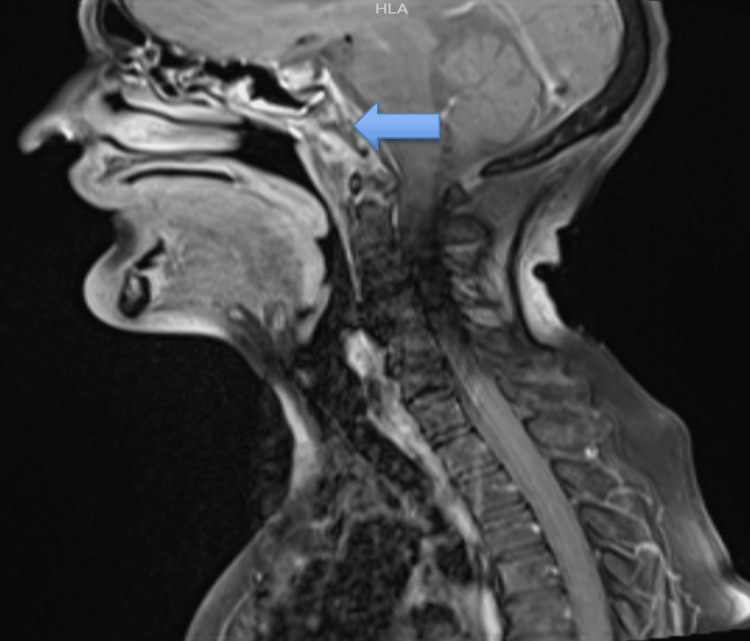
Contrast-enhanced MRI of the brain (T1-weighted) showing post-contrast enhancement of the clivus, and occipital condyles with adjacent peripherally enhancing collection. The blue arrow points to the bone marrow edema in the clivus with diffuse surrounding soft tissue swelling.

**Figure 2:**
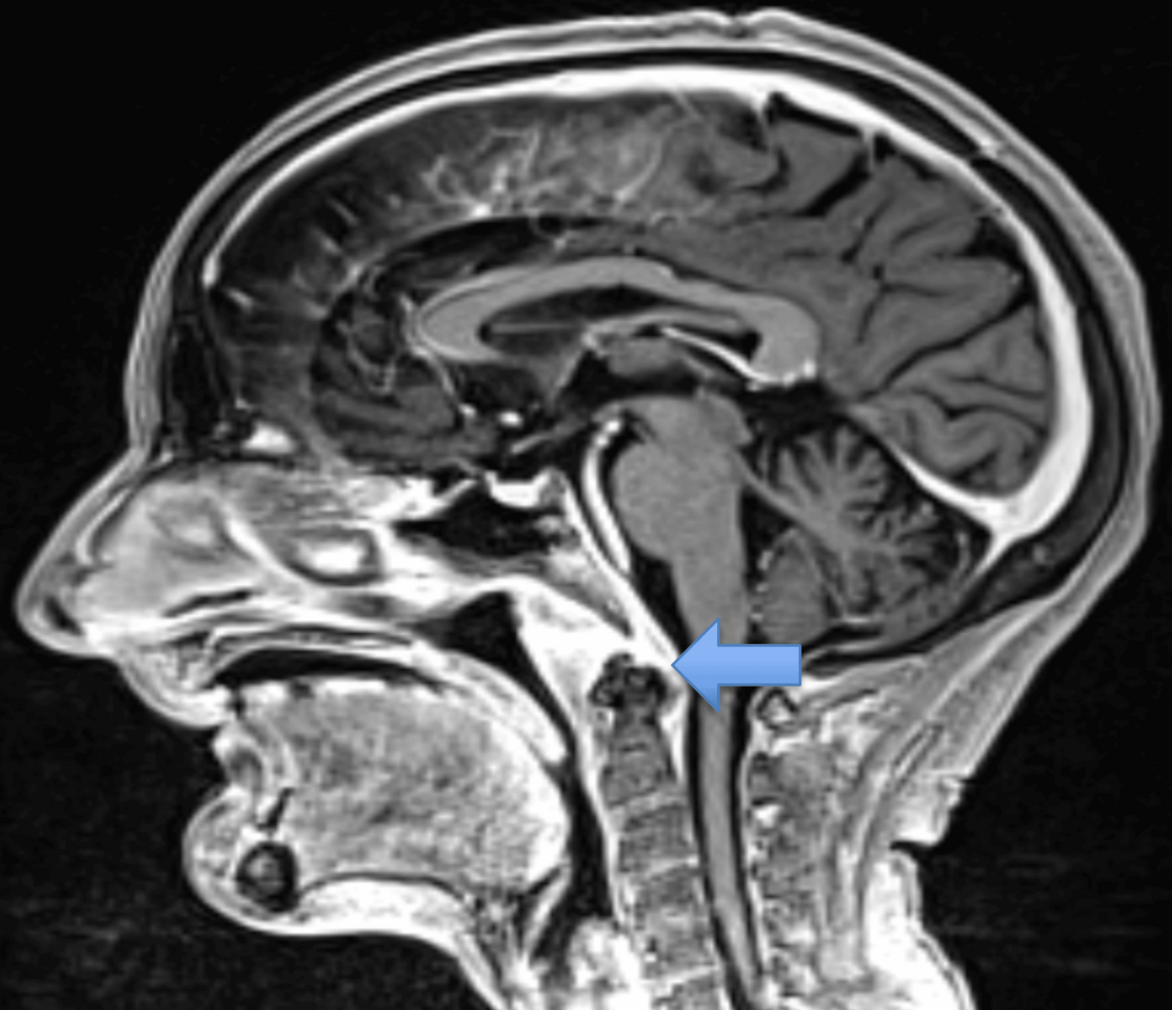
Contrast-enhanced MRI of the brain (T1-weighted, sagittal section) showing post-contrast enhancement of the C1 vertebra with degenerative changes and surrounding soft tissue enhancement. The blue arrow points to the degenerative changes in the C1 vertebra with surrounding soft tissue swelling and collection.

Investigations showed hemoglobin of 12.2 g/dL, a total leukocyte count (TLC) of 11,800/microliter, and a platelet count of 300,000/microliter. Liver and kidney function tests were within normal limits. The erythrocyte sedimentation rate (ESR) was 119 mm/h, and CRP was greater than 10 mg/dL. Her Mantoux test was positive. Chest X-ray was grossly normal. A cervical spine X-ray suggested straightening of the curvature. Contrast-enhanced MRI (CEMRI) of the brain and neck (Figures 1, 2) showed subcortical and periventricular white matter ischemic changes, bone marrow edema in the clivus, occipital condyle, and C1 vertebra, along with diffuse surrounding soft tissue swelling and minimal fluid collection with osseous enhancement. The differential diagnoses included skull base malignancy, chronic infections such as tuberculosis, and post-inflammatory conditions. Lumbar puncture studies showed 80 cells/microliter with a lymphocytic predominance (70%), a glucose level of 159 mg/dL, a protein level of 89 mg/dL, and an adenosine deaminase (ADA) level of 1.4 U/L. Gram stain and culture were negative, herpes simplex virus (HSV) polymerase chain reaction (PCR) and varicella-zoster virus (VZV) PCR were negative, India ink preparation was negative, potassium hydroxide (KOH) for fungus was negative, Truenat for *Mycobacterium tuberculosis* (MTB) DNA was negative, Ziehl-Neelsen (ZN) staining for acid-fast bacilli was negative, and CSF for malignant cells was also negative. Direct laryngoscopy revealed congested and bulky arytenoids.

Treatment

Based on a prolonged history, elevated ESR, CSF findings, a positive Mantoux test, CEMRI findings, and the endemicity of tuberculosis in India, the patient was provisionally diagnosed with tuberculosis of the base of the skull. The patient was empirically managed with antitubercular therapy (isoniazid, rifampicin, pyrazinamide, and ethambutol) along with a tapering dose of dexamethasone for eight weeks. At follow-up after two months, her hoarseness of voice, dysphagia, and slurred speech had improved drastically. The patient remains under follow-up to complete 12 months of antitubercular therapy.

## Discussion

The glossopharyngeal nerve arises from the posterior lateral sulcus of the medulla oblongata, dorsal to the inferior olive, and is closely associated with the vagus and bulbar fibers of the spinal accessory nerve. These three nerves travel together through the jugular foramen. Lesions in the jugular foramen, such as glomus jugulare tumors and basal skull fractures, can damage cranial nerves IX, X, and XI, leading to Vernet syndrome, also known as jugular foramen syndrome. This syndrome is characterized by hoarseness due to vocal cord paralysis (X), difficulty swallowing (IX), and weakness and atrophy of the trapezius and sternocleidomastoid muscles (XI).

The hypoglossal nerve emerges from the medulla in the preolivary sulcus, between the inferior olivary complex and the pyramid, as 10-12 rootlets that are medial to cranial nerves IX, X, and XI. These rootlets merge into two bundles that pass through the dura mater and the hypoglossal canal of the skull [1]. When Vernet syndrome is combined with hypoglossal nerve palsy, it results in "condylar jugular syndrome," also known as "Collet-Sicard syndrome" [2]. Villaret syndrome is characterized by the involvement of cranial nerves IX, X, XI, and XII, along with the cervical sympathetic pathway [3]. In the case reported, the patient exhibited signs of compromised nerves IX, X, and XI, as well as paralysis of half of the tongue due to hypoglossal nerve (XII) involvement. These signs suggested the diagnosis of Collet-Sicard syndrome. The absence of Horner’s syndrome (characterized by ptosis, miosis, enophthalmos, and anhidrosis) ruled out Villaret syndrome. The patient's uncontrolled diabetes likely contributed to the progression of the infectious disease.

It is important to note that besides tuberculosis, other conditions such as tumors, metastases, infections, osteomyelitis, venous thrombosis, arterial dissection, and fractures can also cause these syndromes. Some cases remain idiopathic. In our case, cranial nerve injury was not due to brain ischemia or cervicomedullary junction involvement. Instead, unilateral bone marrow edema and soft tissue swelling in the clivus, occipital condyle, and C1 vertebra, along with infiltration of the lower cranial nerves, led to Collet-Sicard syndrome. Hsu et al. observed Collet-Sicard syndrome in a patient with a C1 fracture combined with congenital basilar impression, noting that lower cranial nerves are susceptible to trauma involving the C1 vertebra [4].

Multiple cranial nerve palsies often pose a diagnostic challenge because nerves can be affected at various points along their course. Lower cranial nerve palsies may result from nerve compression, rootlet avulsion, or stretching. Imaging studies complement physical examination by providing a more accurate assessment of lesion size, location, bony involvement, intracranial extension, and regional nodal disease. CT and MRI are typically the preferred diagnostic tools [5]. Other infectious causes of lower cranial nerve palsies, including HSV, HIV, and varicella-zoster virus, have been reported [6]. Kahane et al. described a patient with jugular foramen syndrome presenting with a palatopharyngeal herpetic eruption, aseptic meningitis, and a high level of serum antibodies to varicella-zoster [7]. Kondo et al. reported a case of herpes zoster infection causing meningoencephalitis and palsies of cranial nerves IX to XI [8]. Our patient tested negative for these infectious diseases and was diagnosed with tuberculosis based on clinical and radiological findings.

The management of Collet-Sicard syndrome involves several approaches, including conservative treatment, medical management, or surgical intervention. The clinical findings underscore the importance of early and accurate diagnosis, as they guide targeted treatment strategies and have significant implications for the patient’s prognosis, potentially improving outcomes and quality of life. Considering the high prevalence of tuberculosis in India along with clinical correlation, our patient empirically received antitubercular therapy and dexamethasone, along with neuromuscular and vocal rehabilitation, resulting in substantial clinical improvement after eight weeks of follow-up. The resolution of edema with steroids led to a rapid alleviation of symptoms.

## Conclusions

This case highlights the rare occurrence of Collet-Sicard syndrome likely due to tuberculosis, a condition that has not been previously documented. Tuberculosis affecting the skull base can lead to multiple cranial nerve palsies, underscoring the need for clinicians to consider infectious causes, particularly in regions where tuberculosis is prevalent. Early diagnosis, aided by clinical suspicion, elevated inflammatory markers, and imaging, is crucial for initiating appropriate treatment and improving patient outcomes. Our patient demonstrated significant recovery following the prompt initiation of antitubercular therapy and steroids, emphasizing the importance of timely intervention. This case also reinforces the necessity of considering tuberculosis in the differential diagnosis of cranial nerve palsies, especially in patients with risk factors such as poorly controlled diabetes.
